# Supplementary material for: Heparin-based hydrogel scaffolding alters the transcriptomic profile and increases the chemoresistance of MDA-MB-231 triple-negative breast cancer cells
Source: Biomater Sci. 2020 Feb 13;8(10):2786–96. doi: 10.1039/c9bm01481k (PMC7497406; doi:10.1039/c9bm01481k)
Supplement: Supplementary file 2 [file BM-008-C9BM01481K-s002.zip › Supplementary File 4/HP-B hydrogel + EGF (s)vControl/Pathways/my_analysis.Gsea.1545201170987/HALLMARK_KRAS_SIGNALING_UP.html]

Details for gene set HALLMARK\_KRAS\_SIGNALING\_UP[GSEA]

|  || Dataset | expr.class.cls#HYDEGF\_versus\_CONTROL.class.cls#HYDEGF\_versus\_CONTROL\_repos |
| Phenotype | class.cls#HYDEGF\_versus\_CONTROL\_repos |
| Upregulated in class | CONTROL |
| GeneSet | HALLMARK\_KRAS\_SIGNALING\_UP |
| Enrichment Score (ES) | -0.451583 |
| Normalized Enrichment Score (NES) | -2.061308 |
| Nominal p-value | 0.0 |
| FDR q-value | 0.0 |
| FWER p-Value | 0.0 |
Table: GSEA Results Summary

  

Fig 1: Enrichment plot: HALLMARK\_KRAS\_SIGNALING\_UP      
 Profile of the Running ES Score & Positions of GeneSet Members on the Rank Ordered List

  

| PROBE | DESCRIPTION (from dataset) | GENE SYMBOL | GENE\_TITLE | RANK IN GENE LIST | RANK METRIC SCORE | RUNNING ES | CORE ENRICHMENT || 1 | EPHB2 | na |  |  | 251 | 2.463 | 0.0055 | No |
| 2 | ENG | na |  |  | 401 | 2.231 | 0.0146 | No |
| 3 | DOCK2 | na |  |  | 500 | 2.120 | 0.0256 | No |
| 4 | SCG5 | na |  |  | 904 | 1.747 | 0.0177 | No |
| 5 | PPP1R15A | na |  |  | 1051 | 1.653 | 0.0226 | No |
| 6 | TPH1 | na |  |  | 1088 | 1.630 | 0.0330 | No |
| 7 | ID2 | na |  |  | 1377 | 1.479 | 0.0291 | No |
| 8 | ITGBL1 | na |  |  | 2075 | 1.245 | 0.0020 | No |
| 9 | SCN1B | na |  |  | 2225 | 1.204 | 0.0033 | No |
| 10 | RABGAP1L | na |  |  | 2717 | 1.076 | -0.0143 | No |
| 11 | AKT2 | na |  |  | 2780 | 1.056 | -0.0095 | No |
| 12 | WDR33 | na |  |  | 2909 | 1.024 | -0.0085 | No |
| 13 | GADD45G | na |  |  | 2933 | 1.019 | -0.0019 | No |
| 14 | CSF2RA | na |  |  | 3308 | 0.942 | -0.0144 | No |
| 15 | CBX8 | na |  |  | 3673 | 0.871 | -0.0269 | No |
| 16 | LAT2 | na |  |  | 3981 | 0.815 | -0.0368 | No |
| 17 | NIN | na |  |  | 4033 | 0.806 | -0.0334 | No |
| 18 | GALNT3 | na |  |  | 4120 | 0.794 | -0.0319 | No |
| 19 | ANGPTL4 | na |  |  | 4183 | 0.784 | -0.0292 | No |
| 20 | RBM4 | na |  |  | 4815 | 0.684 | -0.0571 | No |
| 21 | IGF2 | na |  |  | 5138 | 0.630 | -0.0692 | No |
| 22 | LIF | na |  |  | 5147 | 0.629 | -0.0649 | No |
| 23 | PLAU | na |  |  | 5696 | 0.547 | -0.0895 | No |
| 24 | IL7R | na |  |  | 6375 | 0.455 | -0.1216 | No |
| 25 | F13A1 | na |  |  | 6398 | 0.450 | -0.1193 | No |
| 26 | SEMA3B | na |  |  | 6755 | 0.403 | -0.1350 | No |
| 27 | DNMBP | na |  |  | 7062 | 0.364 | -0.1483 | No |
| 28 | SNAP91 | na |  |  | 7332 | 0.324 | -0.1599 | No |
| 29 | AVL9 | na |  |  | 7544 | 0.299 | -0.1687 | No |
| 30 | ALDH1A2 | na |  |  | 7693 | 0.280 | -0.1744 | No |
| 31 | SDCCAG8 | na |  |  | 7787 | 0.265 | -0.1772 | No |
| 32 | KLF4 | na |  |  | 7914 | 0.254 | -0.1819 | No |
| 33 | EVI5 | na |  |  | 7974 | 0.246 | -0.1831 | No |
| 34 | PDCD1LG2 | na |  |  | 8005 | 0.243 | -0.1829 | No |
| 35 | CD37 | na |  |  | 8032 | 0.239 | -0.1824 | No |
| 36 | MAFB | na |  |  | 8063 | 0.234 | -0.1822 | No |
| 37 | SPARCL1 | na |  |  | 8142 | 0.230 | -0.1846 | No |
| 38 | HDAC9 | na |  |  | 8291 | 0.210 | -0.1907 | No |
| 39 | ITGB2 | na |  |  | 8519 | 0.184 | -0.2013 | No |
| 40 | ATG10 | na |  |  | 8919 | 0.132 | -0.2212 | No |
| 41 | PTCD2 | na |  |  | 8932 | 0.129 | -0.2208 | No |
| 42 | IL1RL2 | na |  |  | 9179 | 0.105 | -0.2330 | No |
| 43 | PRDM1 | na |  |  | 9257 | 0.095 | -0.2363 | No |
| 44 | TSPAN1 | na |  |  | 9310 | 0.088 | -0.2383 | No |
| 45 | PTBP2 | na |  |  | 9486 | 0.078 | -0.2469 | No |
| 46 | TNFRSF1B | na |  |  | 9814 | 0.033 | -0.2638 | No |
| 47 | RELN | na |  |  | 10026 | 0.017 | -0.2748 | No |
| 48 | AKAP12 | na |  |  | 10058 | 0.011 | -0.2763 | No |
| 49 | ZNF639 | na |  |  | 10086 | 0.008 | -0.2777 | No |
| 50 | HKDC1 | na |  |  | 10206 | -0.008 | -0.2839 | No |
| 51 | TLR8 | na |  |  | 10311 | -0.024 | -0.2892 | No |
| 52 | YRDC | na |  |  | 10328 | -0.026 | -0.2898 | No |
| 53 | HBEGF | na |  |  | 10340 | -0.028 | -0.2902 | No |
| 54 | ADAM17 | na |  |  | 10735 | -0.071 | -0.3103 | No |
| 55 | MAP7 | na |  |  | 10826 | -0.082 | -0.3144 | No |
| 56 | GPNMB | na |  |  | 11112 | -0.118 | -0.3285 | No |
| 57 | RGS16 | na |  |  | 11341 | -0.139 | -0.3394 | No |
| 58 | BTBD3 | na |  |  | 11495 | -0.163 | -0.3462 | No |
| 59 | CBL | na |  |  | 11708 | -0.187 | -0.3559 | No |
| 60 | AMMECR1 | na |  |  | 12015 | -0.219 | -0.3703 | No |
| 61 | CFH | na |  |  | 12170 | -0.240 | -0.3765 | No |
| 62 | CDADC1 | na |  |  | 12451 | -0.279 | -0.3891 | No |
| 63 | ZNF277 | na |  |  | 12633 | -0.302 | -0.3963 | No |
| 64 | CROT | na |  |  | 13059 | -0.351 | -0.4159 | No |
| 65 | ADAM8 | na |  |  | 13170 | -0.365 | -0.4190 | No |
| 66 | GNG11 | na |  |  | 13175 | -0.365 | -0.4164 | No |
| 67 | STRN | na |  |  | 13187 | -0.367 | -0.4142 | No |
| 68 | ST6GAL1 | na |  |  | 13237 | -0.376 | -0.4139 | No |
| 69 | ETV4 | na |  |  | 13360 | -0.394 | -0.4173 | No |
| 70 | MAP3K1 | na |  |  | 13524 | -0.417 | -0.4227 | No |
| 71 | FBXO4 | na |  |  | 13594 | -0.423 | -0.4231 | No |
| 72 | ANKH | na |  |  | 13596 | -0.424 | -0.4199 | No |
| 73 | KCNN4 | na |  |  | 13843 | -0.465 | -0.4293 | No |
| 74 | PCSK1N | na |  |  | 13873 | -0.470 | -0.4273 | No |
| 75 | PLAUR | na |  |  | 14166 | -0.503 | -0.4388 | No |
| 76 | G0S2 | na |  |  | 14238 | -0.511 | -0.4386 | No |
| 77 | SPRY2 | na |  |  | 14368 | -0.532 | -0.4414 | No |
| 78 | MTMR10 | na |  |  | 14497 | -0.557 | -0.4439 | No |
| 79 | CCND2 | na |  |  | 14645 | -0.580 | -0.4472 | Yes |
| 80 | INHBA | na |  |  | 14670 | -0.585 | -0.4440 | Yes |
| 81 | PTPRR | na |  |  | 14717 | -0.591 | -0.4419 | Yes |
| 82 | ANO1 | na |  |  | 14752 | -0.598 | -0.4392 | Yes |
| 83 | CXCL10 | na |  |  | 14952 | -0.628 | -0.4449 | Yes |
| 84 | TMEM158 | na |  |  | 14996 | -0.632 | -0.4423 | Yes |
| 85 | ETS1 | na |  |  | 15068 | -0.646 | -0.4411 | Yes |
| 86 | SNAP25 | na |  |  | 15160 | -0.662 | -0.4409 | Yes |
| 87 | JUP | na |  |  | 15173 | -0.664 | -0.4365 | Yes |
| 88 | TSPAN7 | na |  |  | 15243 | -0.677 | -0.4350 | Yes |
| 89 | TSPAN13 | na |  |  | 15319 | -0.691 | -0.4336 | Yes |
| 90 | F2RL1 | na |  |  | 15344 | -0.696 | -0.4296 | Yes |
| 91 | TRIB1 | na |  |  | 15420 | -0.704 | -0.4282 | Yes |
| 92 | TOR1AIP2 | na |  |  | 15659 | -0.755 | -0.4350 | Yes |
| 93 | USP12 | na |  |  | 15853 | -0.792 | -0.4391 | Yes |
| 94 | MMP11 | na |  |  | 15856 | -0.793 | -0.4332 | Yes |
| 95 | SOX9 | na |  |  | 15982 | -0.815 | -0.4336 | Yes |
| 96 | CSF2 | na |  |  | 16092 | -0.842 | -0.4329 | Yes |
| 97 | PRKG2 | na |  |  | 16292 | -0.896 | -0.4365 | Yes |
| 98 | ALDH1A3 | na |  |  | 16296 | -0.897 | -0.4299 | Yes |
| 99 | LAPTM5 | na |  |  | 16354 | -0.911 | -0.4260 | Yes |
| 100 | CBR4 | na |  |  | 16505 | -0.949 | -0.4266 | Yes |
| 101 | ANXA10 | na |  |  | 16535 | -0.953 | -0.4209 | Yes |
| 102 | TRIB2 | na |  |  | 16732 | -1.006 | -0.4236 | Yes |
| 103 | MALL | na |  |  | 16773 | -1.017 | -0.4180 | Yes |
| 104 | CFB | na |  |  | 16882 | -1.049 | -0.4157 | Yes |
| 105 | NGF | na |  |  | 16886 | -1.050 | -0.4079 | Yes |
| 106 | PLAT | na |  |  | 16888 | -1.050 | -0.3999 | Yes |
| 107 | TNFAIP3 | na |  |  | 16896 | -1.053 | -0.3923 | Yes |
| 108 | TRAF1 | na |  |  | 16954 | -1.073 | -0.3872 | Yes |
| 109 | MPZL2 | na |  |  | 17051 | -1.103 | -0.3838 | Yes |
| 110 | BTC | na |  |  | 17061 | -1.103 | -0.3759 | Yes |
| 111 | CXCR4 | na |  |  | 17068 | -1.103 | -0.3679 | Yes |
| 112 | ETV5 | na |  |  | 17111 | -1.116 | -0.3616 | Yes |
| 113 | PLEK2 | na |  |  | 17157 | -1.129 | -0.3554 | Yes |
| 114 | FUCA1 | na |  |  | 17274 | -1.170 | -0.3526 | Yes |
| 115 | NRP1 | na |  |  | 17279 | -1.174 | -0.3439 | Yes |
| 116 | EMP1 | na |  |  | 17346 | -1.196 | -0.3383 | Yes |
| 117 | DCBLD2 | na |  |  | 17441 | -1.232 | -0.3339 | Yes |
| 118 | HIST1H2BB | na |  |  | 17529 | -1.270 | -0.3288 | Yes |
| 119 | CMKLR1 | na |  |  | 17544 | -1.275 | -0.3199 | Yes |
| 120 | PTGS2 | na |  |  | 17994 | -1.483 | -0.3322 | Yes |
| 121 | CAB39L | na |  |  | 18065 | -1.523 | -0.3243 | Yes |
| 122 | GFPT2 | na |  |  | 18069 | -1.525 | -0.3129 | Yes |
| 123 | IL1B | na |  |  | 18115 | -1.556 | -0.3034 | Yes |
| 124 | GPRC5B | na |  |  | 18137 | -1.570 | -0.2926 | Yes |
| 125 | CPE | na |  |  | 18198 | -1.599 | -0.2836 | Yes |
| 126 | PSMB8 | na |  |  | 18223 | -1.614 | -0.2726 | Yes |
| 127 | CCL20 | na |  |  | 18309 | -1.693 | -0.2643 | Yes |
| 128 | CTSS | na |  |  | 18454 | -1.799 | -0.2582 | Yes |
| 129 | BMP2 | na |  |  | 18629 | -1.962 | -0.2524 | Yes |
| 130 | GLRX | na |  |  | 18639 | -1.972 | -0.2379 | Yes |
| 131 | EREG | na |  |  | 18655 | -1.994 | -0.2236 | Yes |
| 132 | ETV1 | na |  |  | 18662 | -2.001 | -0.2087 | Yes |
| 133 | IGFBP3 | na |  |  | 18665 | -2.008 | -0.1936 | Yes |
| 134 | HSD11B1 | na |  |  | 18677 | -2.024 | -0.1788 | Yes |
| 135 | MMD | na |  |  | 18733 | -2.095 | -0.1658 | Yes |
| 136 | BIRC3 | na |  |  | 18942 | -2.435 | -0.1582 | Yes |
| 137 | ITGA2 | na |  |  | 18964 | -2.492 | -0.1404 | Yes |
| 138 | DUSP6 | na |  |  | 18966 | -2.496 | -0.1215 | Yes |
| 139 | SATB1 | na |  |  | 19008 | -2.600 | -0.1039 | Yes |
| 140 | PECAM1 | na |  |  | 19026 | -2.661 | -0.0846 | Yes |
| 141 | TFPI | na |  |  | 19039 | -2.696 | -0.0648 | Yes |
| 142 | PRRX1 | na |  |  | 19101 | -2.909 | -0.0459 | Yes |
| 143 | BPGM | na |  |  | 19126 | -3.042 | -0.0241 | Yes |
| 144 | VWA5A | na |  |  | 19175 | -3.665 | 0.0012 | Yes |
Table: GSEA details [plain text format]

  

Fig 2: HALLMARK\_KRAS\_SIGNALING\_UP      
 Blue-Pink O' Gram in the Space of the Analyzed GeneSet

  

Fig 3: HALLMARK\_KRAS\_SIGNALING\_UP: Random ES distribution      
 Gene set null distribution of ES for **HALLMARK\_KRAS\_SIGNALING\_UP**
